# Live imaging the foreign body response in zebrafish reveals how dampening inflammation reduces fibrosis

**DOI:** 10.1242/jcs.236075

**Published:** 2019-09-26

**Authors:** David B. Gurevich, Kathryn E. French, John D. Collin, Stephen J. Cross, Paul Martin

**Affiliations:** 1 School of Biochemistry, Biomedical Sciences Building, University Walk, University of Bristol, Bristol BS8 1TD, United Kingdom; 2 Department Oral and Maxillofacial Surgery, University Hospitals Bristol, Lower Maudlin Street, Bristol BS1 2LY, UK; 3 Bristol Royal Infirmary, University Hospitals Bristol NHS Trust, Upper Maudlin Street, Bristol BS2 8HW, UK; 4 School of Physiology, Pharmacology and Neuroscience, Biomedical Sciences Building, University Walk, University of Bristol, Bristol BS8 1TD, UK; 5 Wolfson Bioimaging Facility, Faculty of Biomedical Sciences, University Walk, University of Bristol, Bristol BS8 1TD, UK; 6 School of Medicine, UHW Main Building, Heath Park, University of Cardiff, Cardiff, CF14 4XN, UK

**Keywords:** Angiogenesis, Fibrosis, Foreign body, Inflammation, Wound, Zebrafish

## Abstract

Implanting biomaterials in tissues leads to inflammation and a foreign body response (FBR), which can result in rejection. Here, we live image the FBR triggered by surgical suture implantation in a translucent zebrafish model and compare with an acute wound response. We observe inflammation extending from the suture margins, correlating with subsequent avascular and fibrotic encapsulation zones: sutures that induce more inflammation result in increased zones of avascularity and fibrosis. Moreover, we capture macrophages as they fuse to become multinucleate foreign body giant cells (FBGCs) adjacent to the most pro-inflammatory sutures. Genetic and pharmacological dampening of the inflammatory response minimises the FBR (including FBGC generation) and normalises the status of the tissue surrounding these sutures. This model of FBR in adult zebrafish allows us to live image the process and to modulate it in ways that may lead us towards new strategies to ameliorate and circumvent FBR in humans.

This article has an associated First Person interview with the first author of the paper.

## Abstract (precis)

**Summary:** A translucent zebrafish model allows a window on the pathological angiogenic and fibrotic response to suture implantation and how this can be modulated by genetically or pharmacologically dampening inflammation.

## INTRODUCTION

The surgical implantation of medical devices and biomaterials – from cardiac pacemakers to prosthetic joints and surgical sutures – has increased greatly over recent years as technologies advance and the population ages (Major et al., 2015). Once a material is implanted in host tissue, the interactions between the biomaterial and surrounding cells and matrix are critical in determining whether successful integration occurs. In ideal circumstances, biomaterial implantation results in an acute inflammatory response that drives a significant and necessary wound angiogenic response and subsequently limited fibrosis/scarring; this scenario largely recapitulates acute wound healing, and leads to resolution of the repair response and successful biomaterial integration. Failure of biomaterial integration can be due to the exacerbation of the foreign body response (FBR), where acute inflammation transitions to chronic inflammation and is generally accompanied by foreign body giant cell (FBGC) formation and results in fibrous encapsulation (Anderson et al., 2008). This response can limit the efficacy of implantable biomaterials, leading to rejection and adverse outcomes that impact patient quality of life and cause a significant burden on healthcare economics.

Most previous studies of FBR have been performed on mammalian models, such as dogs and mice, largely using histology on fixed samples as an endpoint (Klopfleisch, 2016; Selvig et al., 1998), although more recent intravital studies of implanted plastic chambers in a mouse skin fold model have enabled a degree of dynamic imaging of collagen deposition during FBR using second harmonics (Dondossola et al., 2016). However, these studies are not optimal for high-resolution investigations of the multifaceted and dynamic molecular conversations that occur between tissue and biomaterial; nor can they explain how some materials integrate well with minimal scarring while others undergo an extensive FBR and are ultimately rejected.

We have developed a genetically tractable and translucent model of the FBR that allows for transgenic fluorescent marking of various cells and tissues, enabling the real-time visualisation of immune cell–foreign body interaction over time in a non-invasive manner (Witherel et al., 2017). Aside from external fibrin clot formation, most steps of mammalian wound repair appear to be well conserved in zebrafish and have previously been extensively characterised (Gurevich et al., 2018; Mathias et al., 2009; Renshaw et al., 2006; Richardson et al., 2013); all the initial tissue interactions that are believed to contribute to the development of FBR are known to be present. By fluorescently labelling leukocytes, inflammatory markers and blood vessels in the living organism, we have been able to study the dynamic activities of these lineages in response to the implanted biomaterials, observing the interactions between these cells and the subsequent fibrotic encapsulation, and how these interactions can be modulated to reduce fibrosis and improve integration of biomaterials.

## RESULTS

### The extent of non-resolving scar surrounding the foreign material varies according to suture type

**Fig. 1.**
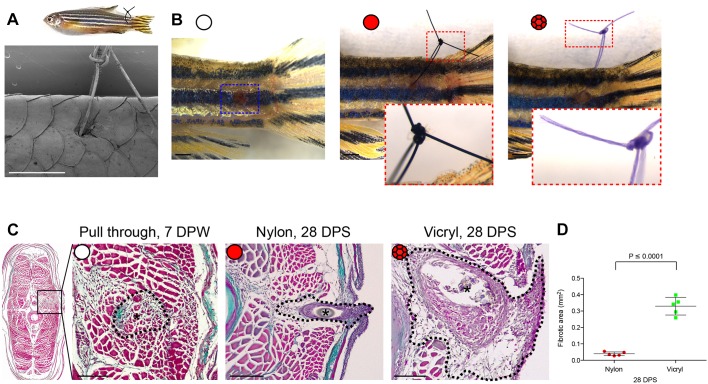
**Extent of foreign body fibrotic encapsulation is dependent on suture type.** (A) Schematic illustration of the zebrafish suture model, with scanning electron micrograph showing a suture in place. *n*=6 independent fish. (B) Representative images of zebrafish following suture pull through (white circle), nylon suture (red circle) or vicryl suture (red braided circle), at 1 day post suturing (DPS) with insets to show suture detail. *n*=5 independent fish per condition. (C) Masson's Trichrome-stained transverse sections of pull through at 7 DPW, and nylon or vicryl sutured fish at 28 DPS, to indicate extent of fibrosis. Sutures are indicated with black asterisks; the zone of scarring and fibrotic encapsulation is indicated by black dotted line overlay. *n*=5 independent fish per condition. (D) Quantification of total area of fibrotic encapsulation, measured from images in C. Error bars in D indicate mean±s.d. Statistical significance is indicated, as determined by two-tailed *t*-test. Scale bars: 1 mm (A,B), 100 μm (C).

Fibrotic encapsulation of foreign bodies, including biomaterials, is a key component of FBR, and is critically important in determining how well a material is integrated into the surrounding tissue (Mikos et al., 1998; Ward, 2008). Previous investigations have shown that materials vary in their biocompatibility, with a consequent variation in degree of inflammatory response and the extent to which fibrosis is induced following implantation (Bryers et al., 2012). To investigate whether zebrafish tissue exhibits a similar variable fibrotic response during FBR to that seen in mammalian tissues, we implanted either 8-0 non-resorbable monofilament nylon or resorbable braided polyglycolic acid (vicryl) sutures of the same dimensions into flank tissue anterior to the base of the tail fin (Fig. 1A,B). Control acute wounds were generated by ‘pulling through’ a vicryl suture at the same location (Fig. 1B). It is already established that, unlike mammalian skin, acute wounds in adult zebrafish skin initially deposit scar collagen but that this subsequently resolves (Richardson et al., 2013). Our Masson's Trichrome histological staining indicates persistent scarring and fibrosis in FBR instances by our endpoint of 28 days post suture implantation (DPS), contrasting with the resolving scarring observed in acute wound repair (Fig. 1C,D; Fig. S1A, and see Richardson et al., 2013). Importantly, the extent of the fibrotic area surrounding vicryl sutures was much larger than the response to nylon sutures (0.3257 mm^2^ compared with 0.0379 mm^2^, Fig. 1D), suggesting that zebrafish tissues react to these materials in similar ways to mammalian tissues.

### All suture types drive an exaggerated and prolonged immune cell response, and the degree of scarring correlates with the extent of inflammatory response

**Fig. 2.**
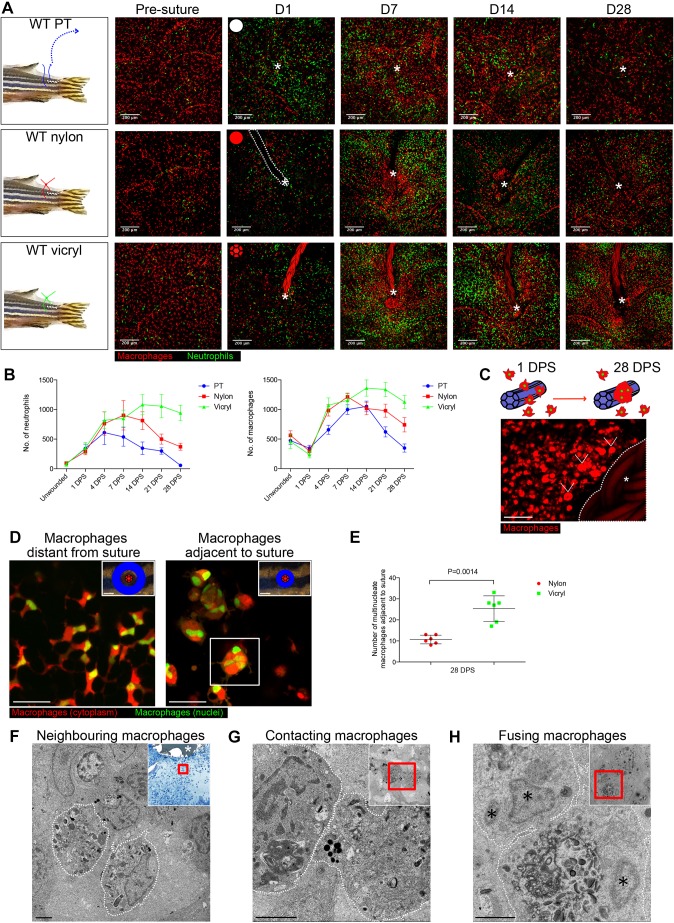
**Magnitude of immune response and numbers of foreign body giant cells are greater for vicryl versus nylon sutures.** (A) Schematic and representative images of Tg(*mpx*:GFP); Tg(*mpeg*:mCherry) double transgenic zebrafish immediately prior to and following suture pull through (PT; top row, white circle), or implantation of nylon (middle row, red circle) or vicryl (bottom row, red braided circle) sutures, at indicated time points. The area of wounding or implantation is marked by a white asterisk; dotted lines indicate nylon suture. *n*=6 independent fish per condition. (B) Quantification of neutrophil and macrophage numbers in the vicinity of wound/suture, measured from images in A. (C) Schematic of macrophage fusion observed in the vicinity of the suture, and representative image of Tg(*mpeg*:mCherry) transgenic adult zebrafish at 28 DPS, showing larger ‘fused’ macrophages, or FBGCs (arrowheads), adjacent to the vicryl suture (white asterisk) with more standard-size macrophages further from the suture. *n*=5 independent fish per condition. (D) Representative images of Tg(*mpeg*:mCherry); Tg(*mpeg*:nlsClover) double transgenic zebrafish at 28 DPS, showing that the larger macrophages adjacent to sutures (within 200 μm radius) are multinucleated (an exemplar such cell, indicated by the boxed area, is revisited in Fig. S2), compared to normal-sized, single-nucleated macrophages at more distal sites (adjacent and ‘distal to suture’ zones illustrated in blue). *n*=6 independent fish per condition. (E) Quantification of images from D, indicating the number of large, multinucleated macrophages adjacent to a wound/suture for nylon and vicryl sutures, at 28 DPS. Statistical significance is indicated, as determined by two-tailed *t*-test. (F–H) Representative electron micrographs showing three different stages of macrophage–macrophage interactions at 28 DPS: (F) nearby but not directly contacting macrophages (membranes indicated by dotted lines); (G) two adjacent macrophages coming into direct plasma membrane contact; (H) a membrane fusion of two macrophages. Insets are low magnification images with red box highlighting the area shown in the high magnification view. In F, the low magnification inset is a Methylene Blue-stained thick section with the suture indicated by a white asterisk. In H, macrophage nuclei are indicated by black asterisks. These panels are also presented without white lines to assist direct observation in Fig. S2C. *n*=4 independent fish. Error bars in B and D indicate mean±s.d. Scale bars: 200 μm (A, insets in D), 50 μm (C), 20 μm (main images in D), 2 μm (F–H).

We next utilised the translucency of the fish to visualise the dynamic interactions underlying the establishment of the FBR. We imaged the same fish and followed the same FBR over extended time periods without the interference of a viewing chamber or other implanted intrusions. Following suture implantation, high-resolution live imaging was used to view Tg(*mpx*:GFP); Tg(*mpeg*:mcherry) double transgenic zebrafish, in which GFP and mCherry mark neutrophils and macrophages, respectively (Ellett et al., 2011; Renshaw et al., 2006). By imaging the same fish at specific time points across the observed 28 days post wounding (DPW) or DPS, we were able to determine the differences in immune cell response to the two suture materials versus acute (pull through sutures) wounding, over time (Fig. 2A). Previous studies examining the FBR have shown that the first immune cells to encounter the biomaterial are neutrophils (Selders et al., 2017), an observation supported by our results. In acute wounds, neutrophil and macrophage numbers peak at 4 DPW and 14 DPW, respectively, after which they resolve back to uninjured levels (Fig. 2B). We see a similar pattern for nylon sutures, although some persistent immune cells remain in the vicinity of the suture at 28 DPS (Fig. 2B). By contrast, we observe a large and persistent immune response up to 28 DPS in vicryl sutured fish, with many immune cells, particularly macrophages, remaining in close proximity to the suture edge (Fig. 2A,B); this immune cell retention appears to correlate with the subsequent extensive fibrosis seen in response to this suture type (Fig. 1C,D).

Close observation of suture-associated macrophages at time points beyond 14 days post implantation indicates that some of these cells appear to be considerably larger than standard macrophages (Fig. 2C). This is suggestive of a phenomenon seen in mammalian FBR and tuberculosis granulomas, where macrophages converge and fuse, transforming into FBGCs as a consequence of chronic inflammation (Davis et al., 2002; Sheikh et al., 2015; ten Harkel et al., 2016). To examine whether a similar response to foreign body-induced chronic inflammation may be occurring here, we used a Tg(*mpeg*:mCherry); Tg(*mpeg*:nlsClover) double transgenic fish to enable visualisation of both cytoplasm and nuclei of macrophages (Fig. 2D). By contrast to pull through injuries, where FBGCs were not detected, we observed a significant number of large, multinucleated macrophages – some with up to six nuclei – in close proximity (within 200 μm) of the suture at 28 DPS, with larger numbers of these FBGCs around vicryl sutures than adjacent to nylon sutures (Fig. 2D,E; Fig. S2A; Movie 1). Capturing the dynamic macrophage fusion events leading to FBGC formation in response to suturing in adult fish proved intractable; however, by following up on our previous studies of localised foreign body implantation in larvae (Witherel et al., 2017) and persistent macrophage stimulation (Gurevich et al., 2018), we were able to visualise this fusion process as it occurred *in vivo* and in its entirety (Fig. S2B; Movie 2). To complement our light microscopy adult studies, we undertook transmission electron microscopy in an attempt to capture the moments when adjacent mononucleate macrophages fuse to generate multinucleated FBGCs (Fig. 2F–H; Fig. S2C). Together, these results indicate that zebrafish immune cells interact with foreign bodies in very similar ways to those observed in mammalian models, and that these interactions may be a key component in directing the extent of fibrotic encapsulation in response to implanted biomaterials.

### The extent of fibrosis reflects immune cell dynamics within the suture-adjacent tissue

**Fig. 3.**
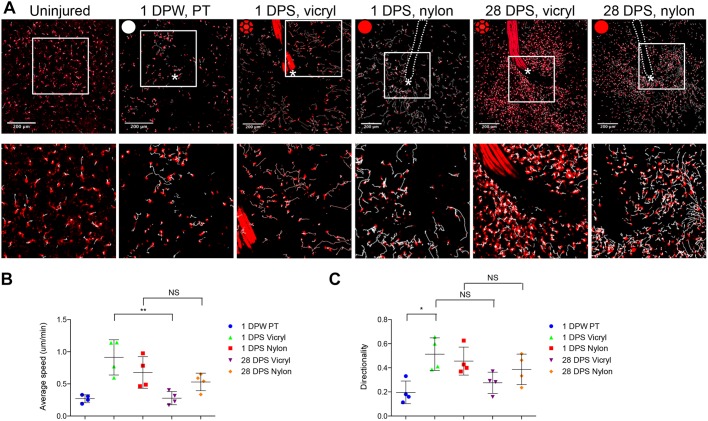
**Immune cell motility and directionality within suture-adjacent tissues are affected by implanted material.** (A) Endpoints from 180 min long representative time lapse movies of Tg(*mpeg*:mCherry) transgenic adult zebrafish at the indicated time points of uninjured, post-pull through (PT, white circle) or suture implant (red and red braided circles for nylon and vicryl, respectively), showing the tracks of macrophages as they respond to the wound/suture. Tracks are generated by automated cell tracking software (see Materials and Methods) and are indicated by white lines; the area of wounding or implantation is marked by a white asterisk; the dotted lines indicates nylon suture; boxed areas shown in lower row. *n*=4 independent fish per condition, per tim epoint. (B) Quantification of mean±s.d. macrophage speed at 1 day and 28 days post implantation, averaged from tracking data, indicating how motility is suppressed at later time points, particularly with vicryl sutures. Statistical significance, as measured by one-way ANOVA, is *P*=0.0045. (C) Quantification of directionality of macrophages at 1 and 28 days post implantation, averaged from tracking data. Statistical significance, as measured by one-way ANOVA, is *P*=0.0078. *P≤0.05; **P≤0.001; NS, not significant. Scale bars: 200 μm.

Having observed the association between extent of inflammation and degree of subsequent scaring at the suture site, we wondered whether live imaging of leukocyte behaviour in response to the suture might provide insight into the development of the FBR. Time lapse photomicroscopy of Tg(*mpeg*:mcherry) unwounded zebrafish flank tissue revealed that macrophages are relatively sparse and static (Movie 3). Following acute wounding, macrophages rapidly migrate towards the wound site; by 1 DPW their motility is still rapid, but their movement once at the wound site lacks directionality (Fig. 3A–C; Movie 4). Suture implantation led to an increase in immune cell directionality at early time points (1 DPS), but a marked reduction in speed at later time points (28 DPS), such that macrophages appear ‘paralysed’ in the tissue adjacent to the suture, particularly in the case of vicryl sutures (Fig. 3B,C; Movies 5–8). This suppression of cell movement extended further from the vicryl suture than for nylon sutures and correlated with the increased extent of fibrosis associated with vicryl sutures (Fig. 1D). These results suggest a causal association between inflammation and localised fibrosis that we might test in our model.

### Tissue inflammation is exacerbated by FBR and induces the formation of an avascular region

**Fig. 4.**
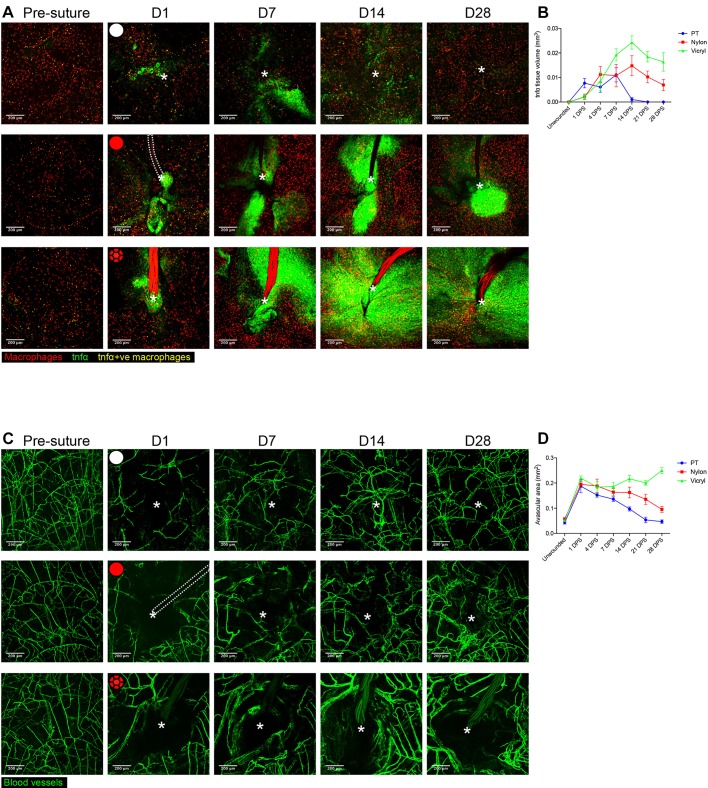
**Extent of *tnfα* expression and size of avascular zone are dependent on suture type.** (A) Representative images of Tg(*tnfα*:GFP); Tg(*mpeg*:mCherry) double transgenic zebrafish immediately prior to and following suture pull through (PT; top row, white circle), or implantation of nylon (middle row, red circle) or vicryl suture (bottom row, red braided circle), at indicated time points, showing macrophages (red), pro-inflammatory macrophages (yellow) and stromal cells expressing *tnfα* in the vicinity of the wound/suture zone (green). The area of wounding or implantation is marked by a white asterisk; dotted lines indicate nylon suture. *n*=8 independent fish per condition. (B) Quantification of the mean±s.d. total inflammatory area surrounding the wound/suture, measured from images in A. (C) Representative images of Tg(*fli*:GFP) transgenic zebrafish immediately prior to and following suture pull through (top row, white circle), or implantation of nylon (middle row, red circle) or vicryl suture (bottom row, red braided circle), to reveal angiogenic response at the indicated timepoints. The area of wounding or implantation is marked by a white asterisk; the dotted lines indicate nylon suture. *n*=8 independent fish per condition. (D) Quantification of the mean±s.d.extent of avascular zone immediately adjacent to wound/suture, measured from images in C. Scale bars: 200 μm.

An effective angiogenic response is pivotal for both wound healing (Eming et al., 2014) and biomaterial integration (Spiller et al., 2015). Our previous work has indicated that pro-inflammatory macrophages expressing tumour necrosis factor α (TNFα) are critical in driving sprouting angiogenesis during tissue repair, but that macrophages must switch to an anti-inflammatory, *tnfα*-negative state at later stages to enable appropriate subsequent vessel remodelling and regression (Gurevich et al., 2018). The Tg(*tnfα*:GFP) transgenic line has previously been used to identify the pro-inflammatory state of cell lineages other than leukocytes including intestinal epithelial cells during inflammatory bowel disease (Marjoram et al., 2015). We used our suture implantation model to observe the dynamic changes that occur with respect to both tissue inflammation and angiogenesis during a FBR. We combined the Tg(*tnfα*:GFP) transgenic line, which marks pro-inflammatory cells, with the Tg(*mpeg*:mCherry) macrophage marker line to reveal macrophages with pro-inflammatory or anti-inflammatory phenotypes. Acute (pull through suture) wounds and both suture types showed increased numbers of pro-inflammatory *tnfα*-positive macrophages within the first two weeks following insult, with numbers of these cells resolving back to uninjured levels by 28 DPW in acute wounds and nylon sutures, but persisting in the case of vicryl sutures (Fig. 4A; Fig. S3A). Acute wounding (pull through sutures) further reveals that the broader wound *tnfα* expression response is also transient, peaking at 7 DPW, being largely resolved by 14 DPW and entirely resolved by 28 DPW (Fig. 4A,B), as previously described for acute wounds (Gurevich et al., 2018; Hübner et al., 1996; MacLeod and Mansbridge, 2016). By contrast, both nylon and vicryl sutures maintain a significant level of *tnfα* expression in the vicinity of the foreign body throughout the observed 28 days (Fig. 4A,B). The overall *tnfα* response induced by vicryl sutures extends out to a much larger area compared with the nylon suture, indicating that tissue inflammation varies with respect to the nature of the implanted material. It should be noted that *tnfα*:GFP can persist for some hours, but we have previously described wound macrophages toggling between *tnfα*-postive and *tnfα*-negative states in less than 15 h (Gurevich et al., 2018).

To examine the angiogenic response to suture implantation, we utilised the Tg(*fli*:GFP) transgenic line, which marks all blood vessels (Lawson and Weinstein, 2002). Acute wounds (pull through sutures) showed a robust revascularisation response, which was well underway by 14 DPW and largely completed by 28 DPW (Fig. 4C,D), in line with the progressive reduction of tissue inflammation (Fig. 4A,B). By contrast, both suture types displayed a reduced capacity to establish blood vessels within close proximity of the implantation site, leading to an avascular zone forming around the suture (Fig. 4C,D). However, the dynamics of this avascular zone differed for the two suture types: nylon sutures appeared to largely re-establish a vascular supply right up to the suture by 28 DPS, whereas vicryl sutures exhibited a progressive increase in the avascular zone, extending out to 350 μm from the suture at 28 DPS. Together, these results suggest a close association between inflammation and subsequently impaired angiogenesis, with the extent of both dependent on the type of implanted biomaterial.

### Dampening the inflammatory response results in reduced fibrosis and improved revascularisation

**Fig. 5.**
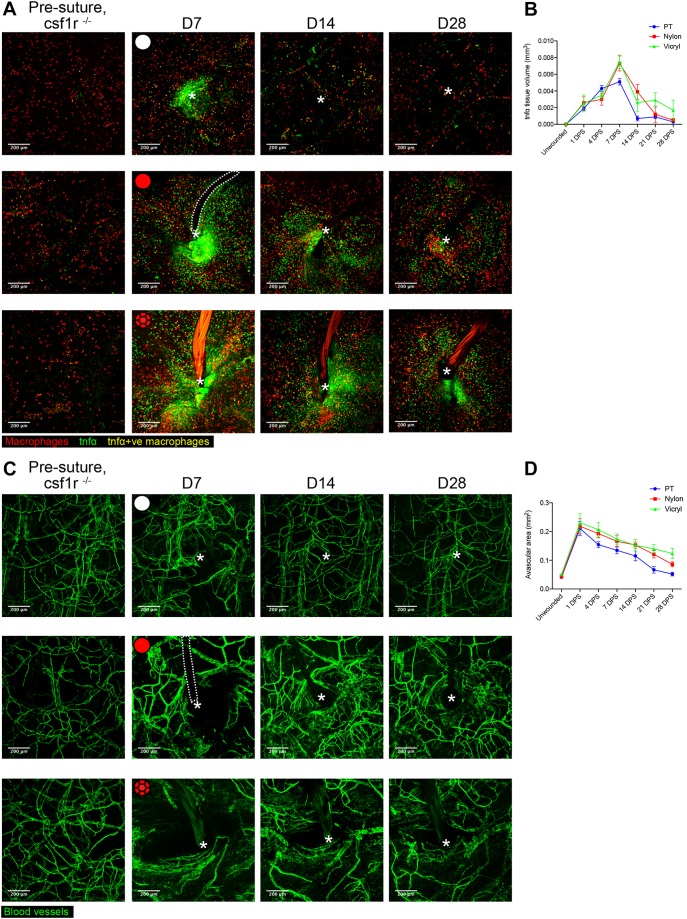
**Genetic immunosuppression results in decreased TNFα expression and reduced avascular zone.** (A) Representative images of *csf1ra*^−/−^ Tg(*tnfα*:GFP); Tg(*mpeg*:mCherry) double transgenic adult zebrafish immediately prior to and following suture pull through (PT, top row, white circle), or implantation of nylon (middle row, red circle) or vicryl suture (bottom row, red braided circle), at indicated time points. The area of wounding or implantation is marked by a white asterisk; the dotted lines indicate nylon suture. *n*=6 independent fish per condition. (B) Quantification of the mean±s.d. altered inflammatory area surrounding the wound/suture, measured from images in A. (C) Representative images of Tg(*fli*:GFP) transgenic adult zebrafish immediately prior to and following suture pull through (top row, white circle), or implantation of nylon suture (middle row, red circle) or vicryl suture (bottom row, red braided circle), at indicated time points. The area of wounding or implantation is marked by a white asterisk; the dotted lines indicate nylon suture. *n*=6 independent fish per condition. (D) Quantification of mean±s.d. avascular zone immediately adjacent to wound/suture, measured from images in C. Scale bars: 200 μm.

Several studies have examined the relationship between extended ‘chronic’ inflammation in the context of impaired healing and how this leads to progressive fibrosis (Morais et al., 2010). To test whether this correlation might be causal in FBR, we next attempted to modulate the inflammatory response to suture implants. A recent investigation into the mechanisms linking these processes in FBR identified colony stimulating factor-1 receptor (CSF-1R) as being part of a central inflammation pathway in macrophages that drives subsequent fibrosis (Doloff et al., 2017). Our first manipulation therefore utilised the zebrafish mutant of this gene, *csf1ra* (referred to as *panther*) that has previously been shown to suppress the normal wound inflammatory response (Gurevich et al., 2018) in combination with the Tg(*tnfα*:GFP); Tg(*mpeg*:mCherry) transgenic line. The *panther* mutant led to a significant ‘rescue’ of the chronic inflammatory state, with reduced expression of *tnfα* in the tissues adjacent to both nylon and vicryl sutures, more closely resembling that of acute pull through wounds at all time points (Fig. 5A,B) rather than the equivalent tissues in wild-type fish (compare to Fig. 4A,B). Combining the *panther* mutant with Tg(*fli*:GFP) revealed a rescue of the avascular zone defect also, with vessels now growing considerably closer to the implanted suture (Fig. 5C,D; compare to Fig. 4C,D).

**Fig. 6.**
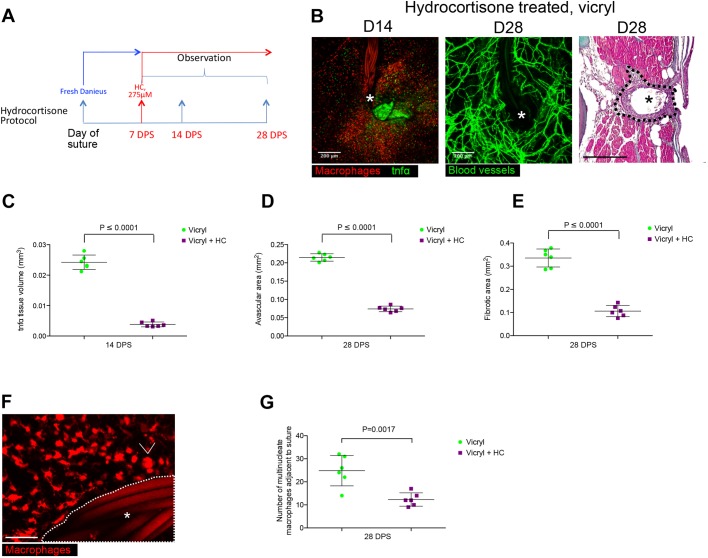
**Pharmacological anti-inflammatory intervention results in a reduced avascular zone, fewer foreign body giant cells and decreased fibrotic encapsulation.** (A) Diagram showing hydrocortisone (HC) treatment protocol used to dampen inflammation during FBR (7–28 DPS). (B) Representative images of Tg(*tnfα*:GFP); Tg(*mpeg*:mCherry), Tg(*fli*:GFP) suture site and Masson's Trichrome-stained section of suture tissue at the indicated time points following vicryl suture implantation and treatment with hydrocortisone. The site of suture implantation is marked by an asterisk; the dotted line indicates the zone of scarring and fibrotic encapsulation. *n*=6 independent fish per condition. (C) Quantification of mean±s.d. total inflammatory area surrounding the wound/suture, measured from images as in B. Statistical significance, as measured by two-tailed *t*-test, is *P*≤0.0001. (D) Quantification of mean±s.d. total area of fibrotic encapsulation, measured from images as in B. Statistical significance, as measured by two-tailed *t*-test, is *P*≤0.0001. (E) Quantification of mean±s.d. avascular zone area immediately adjacent to wound/suture, measured from images as in B. (F) Representative image of Tg(*mpeg*:mCherry) transgenic fish at 28 DPS, showing considerably fewer FBGCs (arrowhead) adjacent to the vicryl suture (white asterisk) following hydrocortisone treatment. *n*=6 independent fish per condition. (G) Quantification of results from images from F, showing that average number of FBGCs adjacent to the vicryl suture (within 200 μm radius) decreases following hydrocortisone treatment. Statistical significance, as measured by two-tailed *t*-test, is *P*=0.0008. Scale bars: 200 μm (B), 50 μm (F).

To complement this genetic approach, we also suppressed inflammation in wild-type fish by treatment with hydrocortisone (from 7 days to 28 days post suture implantation; Fig. 6A), similar to treatment regimens used in previous zebrafish studies (Hasegawa et al., 2017; Richardson et al., 2013). This treatment led to a comparable rescue of FBR and restoration of tissue repair and biomaterial integration to that seen in the *panther* mutant scenario, with much reduced avascular and fibrotic zones (Fig. 6B–E). Furthermore, dampening of inflammation also led to fewer FBGCs and a decrease in the average volume of observed macrophages (Fig. 6F,G; compare to Fig. 2C). Together, these two strategies identify chronic tissue inflammation as a likely candidate for driving the FBR process and, by implication, leading to failure of biomaterial integration, suggesting that the ability to dampen tissue inflammation might be a valuable tool in the amelioration of these problems in a clinical setting.

## DISCUSSION

Our new model of the FBR to suture implantation in zebrafish has allowed us to observe the dynamic interplay of inflammation on cells and tissues including the vasculature and stromal cells that deposit collagen at the implantation site. These studies imply a direct relationship between the extent of the inflammatory response and the degree of fibrotic encapsulation of a foreign body such as a suture and have several implications for the clinic.

### A reciprocal relationship between inflammation, angiogenesis and scarring

Our previous work, as well as that of others, has characterised a dramatic angiogenic response at sites of acute tissue damage. This results in a transient increase in vessel density in the vicinity of the damage site to fuel increased metabolic requirements as the wound heals. These vessels subsequently regress, remodel and normalise back to that seen in uninjured tissue as the repair process finishes (Gurevich et al., 2018; Johnson and Wilgus, 2014). This tightly regulated wound angiogenic response is presumed to be critical because failed angiogenesis associates with chronic, non-healing wounds (Demidova-Rice et al., 2012; Nunan et al., 2014). Interestingly, our current study indicates that biomaterial implantation leads to an avascular zone, which correlates closely with the extent of tissue inflammation, and subsequently also with the zone of fibrotic encapsulation that occurs as a consequence of the FBR. This avascular zone has also been observed in response to implantation of other biomaterials, such as biosensors, and is known to impair the integration and function of such devices (Morais et al., 2010). Indeed, revascularisation post implantation is considered a key element in determining whether a biomaterial integrates or fails (Morais et al., 2010; Yu et al., 2009).

Avascular zones are not an entirely pathological phenomenon; cartilage is avascular, as is the zone beneath the developing epidermis of embryonic skin. Establishment of these avascular territories does not involve inflammation and is believed to be due to presence of avascular glycosaminoglycans such as hyaluronic acid (Feinberg and Beebe, 1983; Hallmann et al., 1987; Martin, 1990). A better understanding of which signals drive the avascular zone in the context of a FBR, and whether they are directly or indirectly released by inflammatory cells, may guide us towards ways for improving vascularity and better tissue integration with implanted biomaterials.

### Modulating the inflammatory cells to regulate angiogenesis and fibrosis

We have previously demonstrated that the pro-inflammatory macrophages that form the first wave of an acute inflammatory response following wounding upregulate vascular endothelial growth factor (VEGF) and are important in driving sprouting angiogenesis (Gurevich et al., 2018); it is also clear that these pro-inflammatory cells induce collagen deposition and fibrosis at repairing wound sites (Cash et al., 2014; Cash and Martin, 2016; Wynn and Barron, 2010). Our current study reveals that implantation of a foreign material triggers a pro-inflammatory macrophage phenotype that largely resolves in all scenarios except for with vicryl sutures, suggesting that these cells may indeed contribute to the increased collagen deposition around foreign body implants. Moreover, implanted foreign materials also increase the extent of general tissue inflammation, which varies in extent depending on the type of material. Intriguingly, this zone of inflammation is closely correlated with both the observed fibrotic encapsulation response and the subsequent avascular zone. We have previously observed this potential link between fibrosis and vascularisation in the context of osteopontin knockdown, with the suppression of this wound inflammatory marker in mouse wounds resulting in reduced scarring, increased angiogenesis and rapid repair (Mori et al., 2008). In our present work, reduction in tissue inflammation by genetic means (*panther* mutant) or chemically (hydrocortisone treatment) appears to decrease both fibrosis and avascularity, suggesting that inflammation might be a critical factor in determining whether the biomaterial will integrate or undergo rejection via FBR, given the association between implant failure, the extent of angiogenesis and fibrosis. Indeed, this is in line with recent attempts to make biomaterials more biocompatible, which have focused on reducing inflammation (Kim et al., 2017; Zhang et al., 2014). However, our results also indicate that pro-inflammatory macrophages are still present in areas immediately adjacent to implanted sutures, particularly up to 14 days post suture implantation; this corresponds with the main vessel sprouting response during the repair and integration process, suggesting that these cells are likely playing a similar important pro-angiogenic role as previously determined (Gurevich et al., 2018). Taken together, our results suggest that future innovations to maximise biomaterial integration should distinguish between pro-inflammatory macrophages and other pro-inflammatory tissues.

### What role for giant cells in the FBR?

FBGCs, which are presumed to be formed by macrophage fusion, were first described 50 years ago (Mariano and Spector, 1974) and are considered a characteristic component of the tissue response to implanted materials as well as to some parasitic infections (Chambers, 1978; Davis et al., 2002). Indeed, activation and aggregation of distinct, specialised macrophages in response to persistent and antagonistic stimuli such as mycobacterium are now thought to be the key events driving the formation of granulomas in response to tuberculosis (McClean and Tobin, 2016; Ramakrishnan, 2012). Our study is the first to dynamically image these cells aggregating in high densities prior to FBGC formation, and to our knowledge the first to visualise the entire macrophage fusion process *in vivo*. Our studies strongly suggest that polynucleated macrophages can arise by fusion, and not only by modified cell divisions (Herrtwich et al., 2016). We note that FBGCs are more commonly seen in the vicinity of vicryl rather than nylon sutures, suggesting that there may be a threshold level for both cell density and the phenotypic state of inflammatory response before fusion will occur. Macrophages that come into direct contact with certain biomaterials are believed to undergo a process of ‘frustrated phagocytosis’, where the inability to engage with the material drives the fusion process; this leads to a subsequent decrease in phagocytic ability and a concomitant increase in free radical, enzyme and acid release to degrade implanted materials (Anderson et al., 2008). However, many questions remain concerning the precise triggers and mechanisms that underlie this fusion process to activate FBGC formation. Our model presents a valuable opportunity for unravelling the dynamic nature of these fusion mechanisms, and gaining insight into what the specific function of FBGCs might be.

### Adult zebrafish as an important new *in vivo* model of FBR and clinical implications

This study represents the first time that the cell and tissue interactions underlying the FBR between biomaterial and surrounding tissue have been imaged dynamically, *in vivo* and non-invasively. The numerous similarities to mammalian FBR marks the zebrafish as a valuable model for increasing our understanding of the cellular and molecular basis for FBR in response to specific biomaterials. Our approach is particularly powerful as it allows the examination of several key processes and cell players – inflammation, formation of FBGCs and the angiogenic response – in the same animal over time, permitting the specific tracking and dissection of dynamic cell–cell conversations. In addition, the imaging opportunities in zebrafish combined with its genetic tractability and amenability for chemical/pharmacological intervention, have allowed us to investigate how modulating inflammation in various ways can impact on tissue restoration during FBR. The advances in our understanding presented here will drive further identification and refinement of methods that alleviate FBR and improve biomaterial integration.

## MATERIALS AND METHODS

### Zebrafish strains and maintenance

All experiments were conducted with approval from the local ethical review committee at the University of Bristol and in accordance with the UK Home Office regulations (Guidance on the Operation of Animals, Scientific Procedures Act, 1986). Wild-type and transgenic lines Tg(*fli1*:eGFP) [referred to as Tg(*fli*:GFP)] (Lawson and Weinstein, 2002), Tg(*mpx*:GFP) (Renshaw et al., 2006), Tg(*mpeg1*:mCherry) [referred to as Tg(*mpeg*:mCherry)] (Ellett et al., 2011), TgBAC(*tnfα*:GFP) [referred to as Tg(*tnfα*:GFP)] (Marjoram et al., 2015) were maintained on a TL wild-type background, and staging and husbandry were performed as previously described (Westerfield, 1995). The mutant strain used was *csf1ra^j4e1^* (Parichy et al., 2000), maintained on an AB background or used in combination with transgenic lines as indicated. *csf1ra^j4e1^* mutants were genotyped by visual inspection for absence of mature xanthophores as previously described (Parichy et al., 2000).

### Suture implantation into adult zebrafish

Adult zebrafish suturing was performed as previously described (Witherel et al., 2017). Briefly, zebrafish were anaesthetised in tank system water with 0.1 mg/ml tricaine (ethyl 3-aminobenzoate methanesulfonate; Sigma-Aldrich, Hamburg, Germany) and subsequently placed onto a foam surgical stand for surgery. Single interrupted sutures were implanted by placing a single loop through the tail, ∼3 mm anterior to the tailfin, using either nylon non-absorbable sutures (polyamide, 8-0 monofilament, 3/8 tapered needle, S&T, Neuhausen, Switzerland) or vicryl, absorbable sutures (polyglactin, 8-0 braided, 3/8 needle, Ethicon, Somerville, NJ). Pull through control wounds were generated by implantation of a suture at the exact same anatomical location, which was immediately ‘pulled through’ and removed.

### Imaging of adult zebrafish

For all imaging experiments, fish were initially anaesthetised in 0.3% Danieau's solution with 0.1 mg/ml tricaine, and subsequently embedded using 1.5% (w/v) agarose added over the tail in a 10 cm petri dish. Care was taken to keep agarose away from the gills. For time lapse imaging, fish were maintained in a lightly anaesthetised state at 0.05 mg/ml tricaine throughout to allow continued breathing; fish that were no longer breathing by the end of movie acquisition were excluded from analysis. Gross anatomical images were generated on a Leica M205 FA system (Leica Microsystems). Confocal images and time lapse movies were generated on a Leica SP8 MP/CLSM system (Leica Microsystems).

### Hydrocortisone treatment

For drug treatments, fish were treated with 275 μM hydrocortisone (Sigma-Aldrich) dissolved in ethanol, as previously described (Richardson et al., 2013); 0.1% absolute ethanol was used for all treatments as well as vehicle control.

### Masson's Trichrome staining

Harvested fish tails were immediately fixed in 4% paraformaldehyde (PFA) overnight at 4°C on a rocker, washed with PBS and then decalcified in 0.5 M EDTA (Sigma-Aldrich, Hamburg, Germany) for 7 days at 4°C on a rocker, replacing the EDTA solution on the third day. Samples were then stained for Masson's Trichrome, as previously described (Witherel et al., 2017).

### Scanning electron microscopy

Whole fish were fixed and processed at 28 DPS as previously described (van den Berg et al., 2019). Samples were prepared using a Leica CPD300 critical point drier, sputter coated with Au/Pd using an Emitech 575X sputter coater, and examined using an FEI Quanta 200FEG SEM.

### Transmission electron microscopy

Tails were harvested at 28 DPS, fixed and processed as previously described (Nunan et al., 2015). Ultrathin (0.02 μm) sections were images on a Tecnai 12-FEI 120 kV BioTwin Spirit transmission electron microscope.

### Needle stick injury and LPS treatment

Needle stick wound induction was performed to the dorsal somites opposite the cloaca with either a 30 G needle (Becton Dickinson), as previously described (Gurevich et al., 2016). As a persistent inflammatory signal, injured fish were immediately exposed to lipopolysacchiride (LPS; Sigma-Aldrich), at a working solution of 100 μg/ml diluted in E3 water. As for adult fish, confocal images were generated on a Leica SP8 MP/CLSM system (Leica Microsystems).

### Image analysis

All image analysis was performed in ImageJ. Detection, tracking and spatial analysis of immune cells used the Modular Image Analysis automated workflow plugin for Fiji (Cross, 2019, https://zenodo.org/record/2628332; Rueden et al., 2017; Schindelin et al., 2012). Sample motion due to tissue growth was corrected using translation-based registration via the SIFT Align plugin for Fiji (Lowe, 2004; Saalfeld, 2008, https://github.com/axtimwalde/mpicbg) followed by B-spline unwarping using the BUnwarpJ plugin (Arganda-Carreras et al., 2006). Cell features were enhanced prior to detection using the WEKA pixel classification plugin (Arganda-Carreras et al., 2017). Noise in the enhanced image was removed using a 3D median filter, and immune cells isolated from background using the Otsu threshold method with a constant user-defined offset (Otsu, 1979). The binarised image was refined using 2D hole filling and a 3D distance-based watershed transform (Legland et al., 2016). Immune cells were identified in 3D as contiguous regions of pixels labelled as foreground using the MorphoLibJ plugin (Legland et al., 2016). An outline of the suture was manually annotated, with any immune cells detected coincident with it assumed to correspond to misdetection; these cells were excluded from further analysis. Immune cells were tracked between frames using the Munkres algorithm with scores based on object centroid separation (Munkres, 1957). Track spatial coordinates were used to calculate instantaneous velocity and track orientation in the *xy*-plane. A static reference point corresponding to the suture was manually identified in each video (separate from the previously detected outline). The angle between the instantaneous track orientation and this point was also measured (i.e. an angle of 0° corresponds to a cell moving directly towards the suture).

### Statistical analysis

All statistical analysis was performed using Graphpad Prism. Power calculations (Mann–Whitney *U*-test performed at 90% power at 5% significance) indicated that *n*=6 animals (effect size=2.4) are adequate for each analysed parameter for statistical significance. For several experiments, a lower number of samples proved to have sufficient power for significance. Fish were allocated to treatment groups by simple random sampling. Student's *t*-test were used except in the case of comparisons involving more than two groups; in these instances, one-way ANOVA was performed for all comparisons, and a Bonferroni multiple comparison test was subsequently performed.

## Supplementary Material

Supplementary information
