# Supplementary material for: Live imaging the foreign body response in zebrafish reveals how dampening inflammation reduces fibrosis
Source: J Cell Sci. 2019 Sep 26;133(5):jcs236075. doi: 10.1242/jcs.236075 (PMC6899018; doi:10.1242/jcs.236075)
Supplement: Supplementary information [file joces-133-236075-s1.pdf]

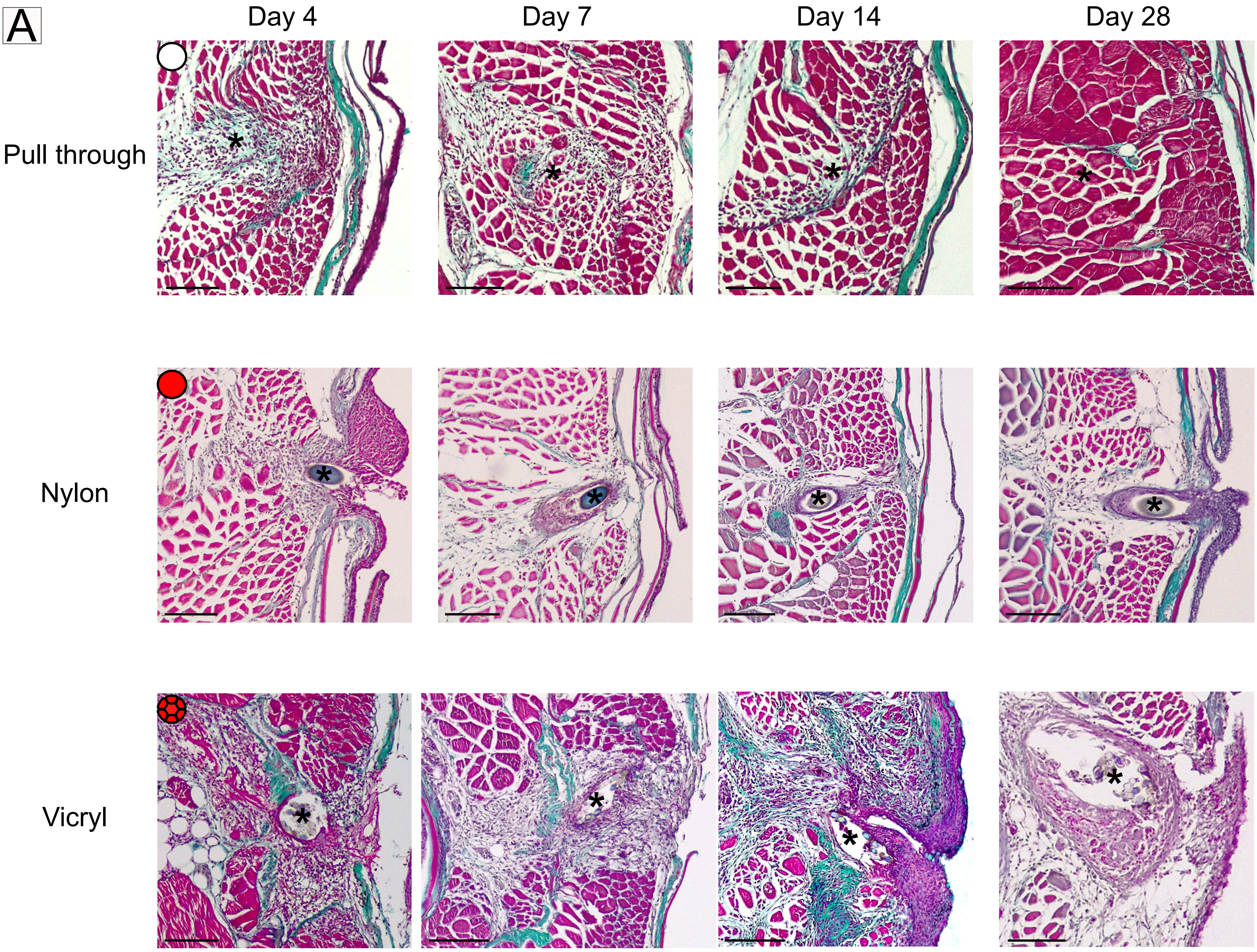

Figure S1: Representative Masson's Trichrome stained transverse sections of pull through, nylon or vicryl sutured fish at 4, 7, 14 and 28 DPS, as indicated. Site of wound or suture indicated with black asterisk. N = 5 independent fish per condition per timepoint. Scale bars = 200μm.

Journal of Cell Science • Supplementary information

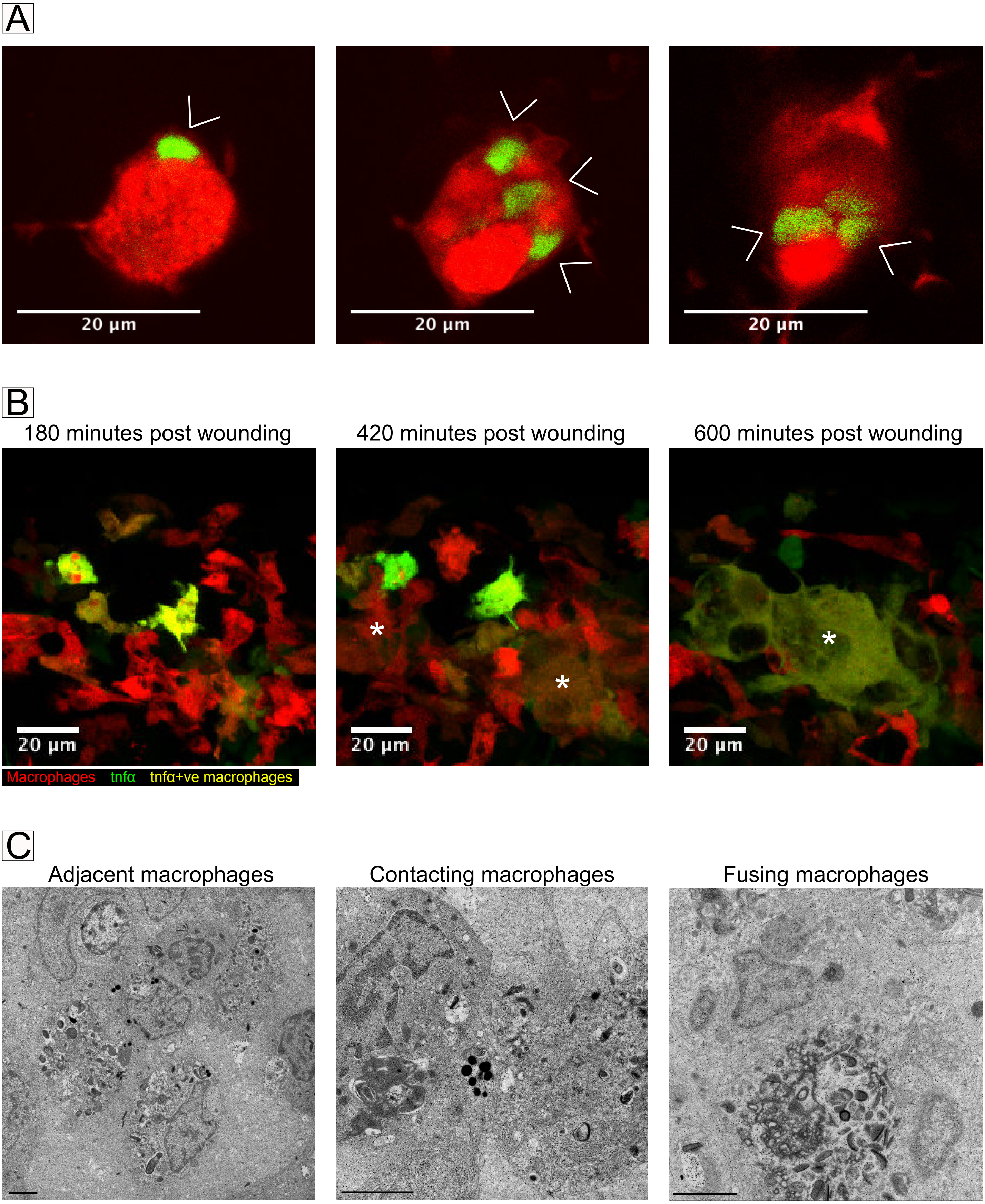

Journal of Cell Science • Supplementary information

Figure S2: A) Several z-stack images from a single “large” macrophage adjacent to a vicryl suture from the image shown in Figure 2D (boxed area), with arrows indicating multiple nuclei (green) within the cell. B) Several stills from Movie 2, showing macrophages in Tg(*mpeg*:mCherry); Tg(*tnfα*:GFP) double transgenic larvae prior to, during, and after fusion at 180, 420 and 600 minutes post injury, in response to persistent inflammatory stimulus. Two *tnfα*:GFP positive cells fuse with a number of *tnfα*:GFP negative macrophages and their GFP becomes diluted throughout the newly fused cell. C) TEM images from Figure 2F-H, without dotted lines and other annotations, to allow for unobstructed examination. Scale bars: C = 2µm.

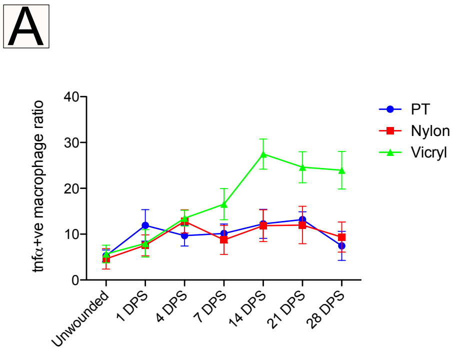

Figure S3: A) Quantification of *tnfα* expressing macrophages as a proportion of total macrophages in the area surrounding the wound/suture, measured from images in Figure 4A.

Journal of Cell Science • Supplementary information

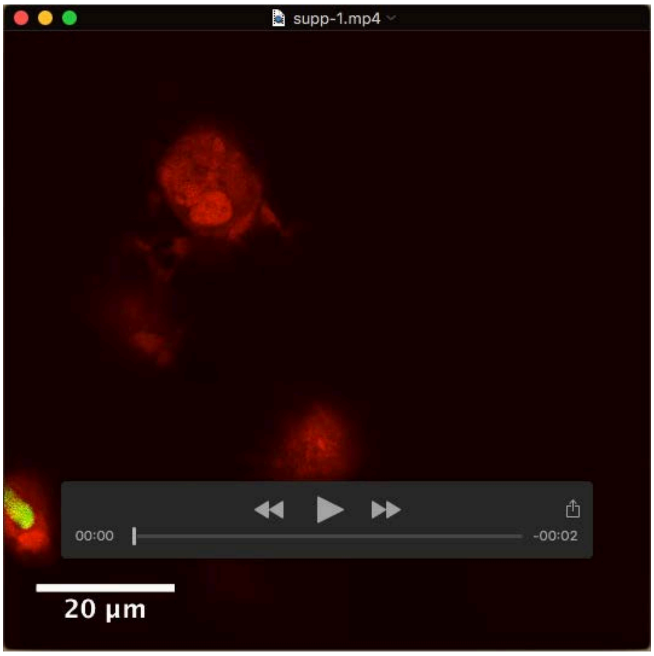

Movie 1: Slice by slice z-stack movie of the cells shown in Figure 2D, identifying numerous macrophages as FBGCs with multiple nuclei. Movie z-step size = 2µm, scale bar = 20µm.

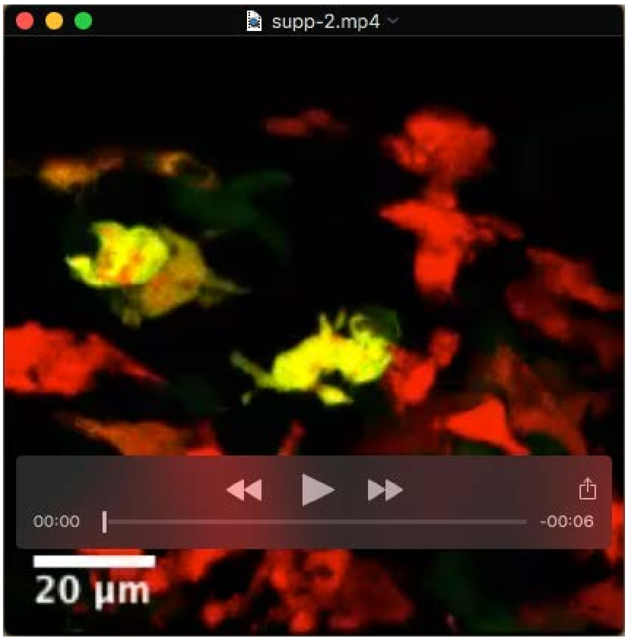

Movie 2: Representative z-projection timelapse movie of the flank of a laterally mounted, wounded Tg(*mpeg*:mCherry); Tg(*tnfa*:GFP) double transgenic, 5 day post fertilisation (dpf) larvae, showing dynamic fusion of macrophages.

Journal of Cell Science • Supplementary information

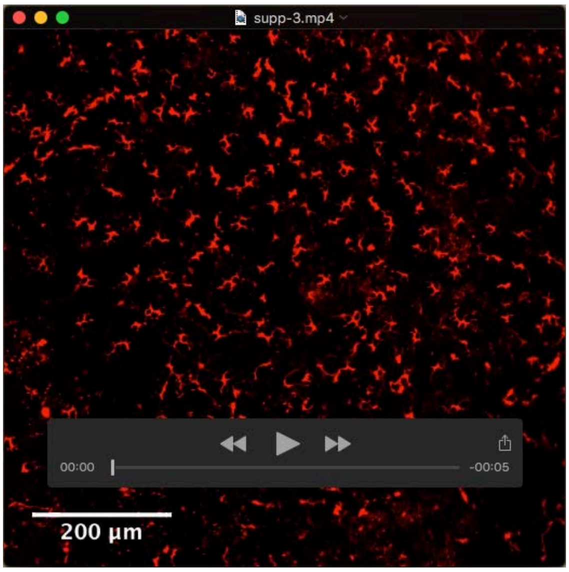

Movie 3: Representative z-projection timelapse movie of the flank of a laterally mounted uninjured Tg(*mpeg*:mCherry) transgenic, showing tracking of macrophage dynamics and ‘patrolling’ behaviour, imaged every 5 minutes for 180 minutes.

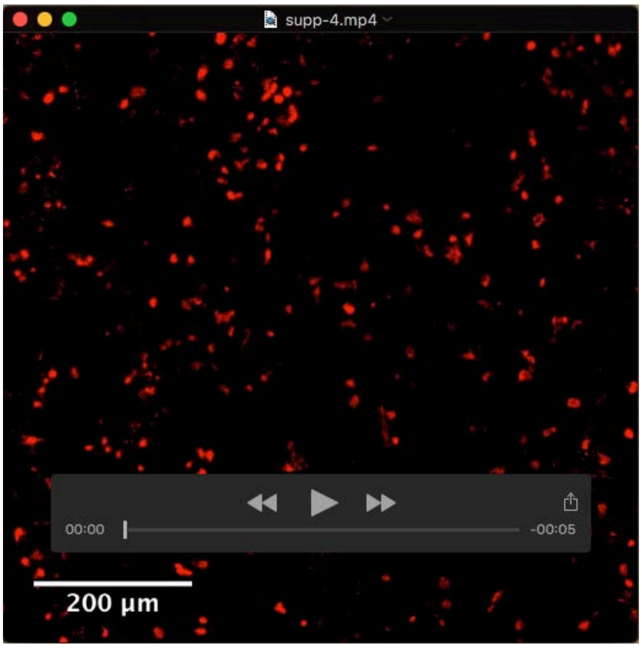

Movie 4: Representative z-projection timelapse movie of the flank of a laterally mounted ‘pull through’ injured Tg(*mpeg*:mCherry) transgenic at 1DPW, showing tracking of macrophage dynamics, imaged every 5 minutes for 180 minutes.

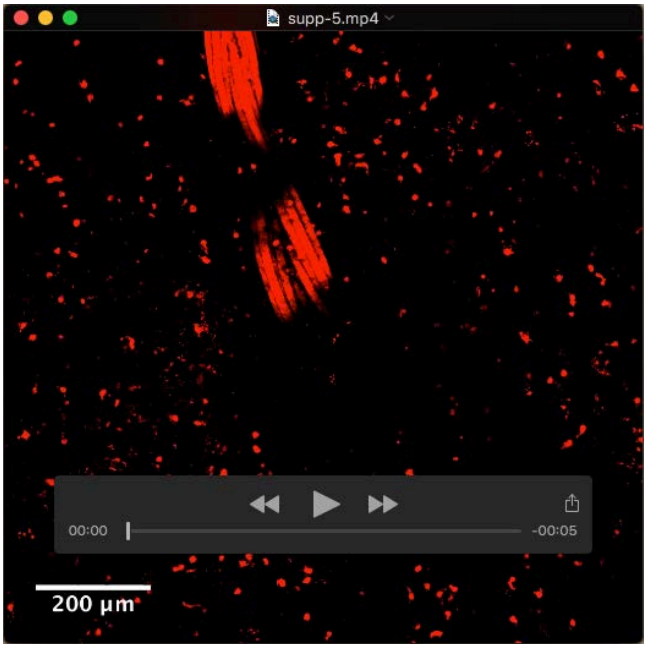

Movie 5: Representative z-projection timelapse movie of the flank of a laterally mounted, vicryl sutured Tg(*mpeg*:mCherry) transgenic, 1DPS, showing tracking of macrophage dynamics, imaged every 5 minutes for 180 minutes.

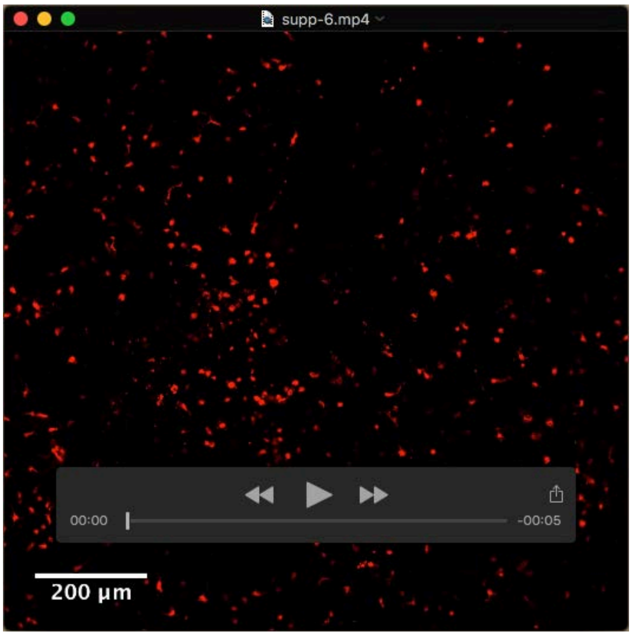

Movie 6: Representative z-projection timelapse movie of the flank of a laterally mounted, nylon sutured Tg(*mpeg*:mCherry) transgenic, 1DPS, showing tracking of macrophage dynamics, imaged every 5 minutes for 180 minutes.

Journal of Cell Science • Supplementary information

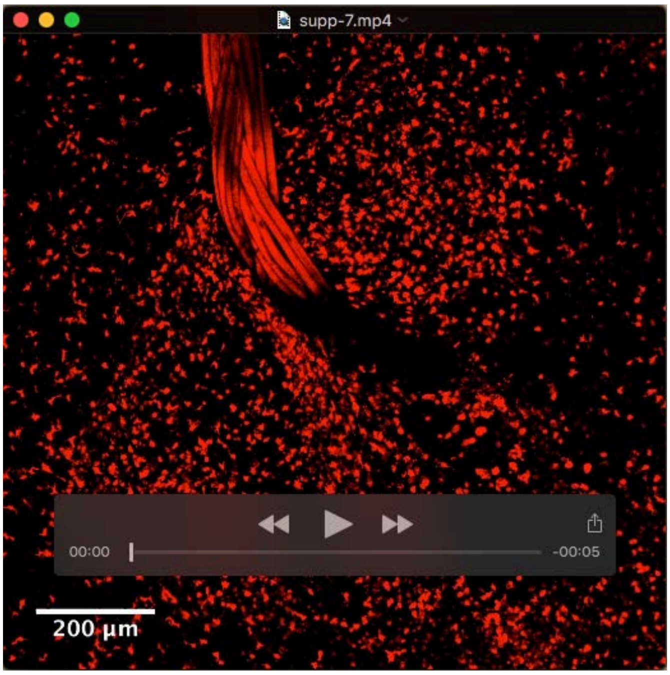

Movie 7: Representative z-projection timelapse movie of the flank of a laterally mounted, vicryl sutured Tg(*mpeg*:mCherry) transgenic, 28DPS, showing tracking of macrophage dynamics, imaged every 5 minutes for 180 minutes.

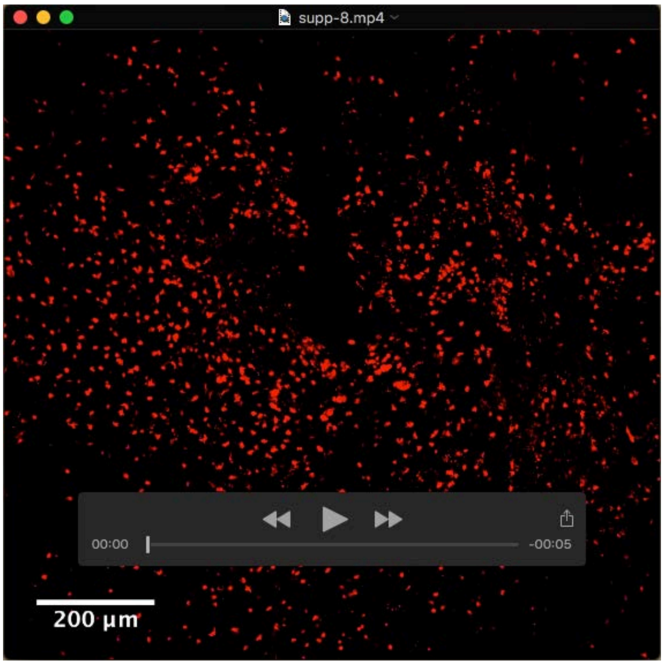

Movie 8: Representative z-projection timelapse movie of the flank of a laterally mounted, nylon sutured Tg(*mpeg*:mCherry) transgenic, 28DPS, showing tracking of macrophage dynamics, imaged every 5 minutes for 180 minutes.
